# Nuclear Export of L-Periaxin, Mediated by Its Nuclear Export Signal in the PDZ Domain

**DOI:** 10.1371/journal.pone.0091953

**Published:** 2014-03-14

**Authors:** Yawei Shi, Lei Zhang, Ting Yang

**Affiliations:** 1 Institute of Biotechnology, Key Laboratory of Chemical Biology and Molecular Engineering of Ministry of Education, Shanxi University, Taiyuan, Shanxi, P. R. China; 2 Institute of Clinical Medicine and Department of Cardiology, Renmin hospital, Hubei University of Medicine, Shiyan, Hubei, P. R. China; University of Toronto, Canada

## Abstract

Myelinating Schwann cells specifically express L-periaxin (L-PRX) in the mammalian peripheral nervous system. Several loss-of-function mutations in periaxin have been described and linked to autosomal recessive Dejerine Sottas neuropathy and to demyelinating Charcot-Marie-Tooth disease. The localization of L-periaxin is developmentally regulated in the nucleus and the plasma membrane of Schwann cells. In this study, L-periaxin, which contains a PDZ domain, a nuclear localization signal (NLS) domain, a repeat domain, and an acidic domain, was localized in the cytoplasm of RSC96 cells. By contrast, a mutant L-periaxin with a deleted PDZ domain was localized mainly in the nucleus of RSC96 cells. After a nuclear cyclin A1, which is localized exclusively in the nucleus, was fused with the PDZ domain, cyclinA1was found in the cytoplasm of RSC96 cells. Treatment with leptomycin B (LMB), a specific inhibitor of nuclear export mediated by leucine-rich nuclear export signal (NES), also causes nuclear accumulation of wild-type L-periaxin. Double leucine mutation (L83, 85Q) in the putative NES in the PDZ domain prevented L-periaxin nuclear export and induced nuclear accumulation. These results suggested that the localization of L-periaxin in the cytoplasm is supported by NES in the PDZ domain.

## Introduction

Periaxin is a non-compact myelin protein in the peripheral nervous system (PNS) [1]. Periaxin gene has been characterized in Dejerine-Sottas [2] and Type 4F Charcot-Marie-Tooth degenerating peripheral neuropathies [3], [4]. L-periaxin has been proposed as a marker of devil facial tumor disease, which is a peripheral nerve sheath neoplasm of Schwann cell origin [5]. Periaxin has two isoforms, namely, L-periaxin (147 kDa) and S-periaxin (16 kDa) generated by alternative mRNA splicing [6]. L-periaxin and S-periaxin contain a PDZ domain at the N-terminus. The PDZ domain, named after post-synaptic density protein (PSD)-95, *Drosophila* discs-large protein (Dlg), and zonula occludens protein (ZO)-1, is a motif containing 80 to 100 amino acids found in single or multiple sets of membrane-bound and cytoplasmic proteins [7]. PDZ domains are involved in linking receptors to effector molecules and in clustering ion channels and receptors via interactions [7], [8]. Similar to other proteins that contain a PDZ domain, L-periaxin is localized in the plasma membrane of myelinating Schwann cells. By contrast, S-periaxin is diffusely expressed in the cytoplasm. L-periaxin stabilizes the dystroglycan glycoprotein complex (DGC) by directly interacting with dystrophin-related protein 2 (DRP2) in a macromolecular complex that may provide a link between the extracellular matrix and the Schwann cell [9], [10]. In contrast to L-periaxin, DRP2 is not a component of Schmidt-Lanterman incisures, or cytoplasm-filled channels in PNS myelin [11]. Quantitative studies have shown that periaxin comprises 16% by weight of PNS myelin protein, whereas DRP2 contains 0.2% [12]. These values are inconsistent with an exclusive stoichiometric relationship between periaxin and Drp2 in a dystroglycan complex, suggesting that periaxin has other functions in addition to PDG complex formation and appositions [11]. L-periaxin forms homodimers via a PDZ domain and clusters PDG complexes in the Schwann cell plasma membrane.In ΔPDZ-PRX mice, it reduced Schwann cell elongation and retarded but not prevented development of normal conduction velocities to a maximum value [13].

L-periaxin is also expressed in lens fibers and exhibits maturation dependent redistribution, clustering discretely at tricelluar junction in mature fiber cells. In the lens, L-periaxin is present as a part of ezrin, periaxin, periplakin, and desmoyokin complex and functions in cell adhesive interactions, which is required for hexagonal geometry and membrane organization of mature lens fibers [14].

L-periaxin protein can be observed in a selected nucleus of Schwann cells as early as embryonic age (E) 14.5 in the mouse sciatic nerve; this finding is consistent with the presence of a nuclear localization signal in a specific protein. However, L-periaxin is mostly associated with the plasma membrane of Schwann cells at E 17.5. Postnatal periaxin expression is further upregulated in myelination-involved cells [9], [15], [16]. In these cells, periaxin expression is initially observed adaxonally and then becomes localized in particular regions associated with the abaxonal Schwann cell membrane as myelination occurs [9], [15]. In other words, the localization of L-periaxin in Schwann cells changes after a spiralization phase of axon ensheathment is completed, this finding has suggested that L-periaxin participates in membrane-protein interactions required to stabilize the mature sheath [15].

These observations have also suggested that the subcellular distributions of L-periaxin are governed by distinct molecular determinants. In this study, a functional nuclear export signal (NES) was identified in the PDZ domain of L-periaxin. We also found that the cytoplasmic localization of L-periaxin was supported by NES. Disruption of NES or treatment with Leptomycin B (LMB) impaired the nuclear export activity and induced accumulation of L-periaxin in the nucleus.

## Materials and Methods

### Plasmid Construction

EGFP-L-periaxin (EGFP-L-PRX), in which the full-length L-periaxin (AB046840.1) was fused with an enhanced green fluorescent protein (EGFP), was generated by PCR with appropriate primer sets. L-periaxin cDNA was used as a template and inserted in *Eco*RI-*Sal*I sites of the pEGFP-C3 expression vector. A similar strategy was performed to generate EGFP fusion constructs with DPDZ-L-PRX (104-1398 aa, EGFP-DPDZ-L-PRX), EGFP-PDZ (18-102 aa), and cyclin A1 (NM_001111045, 1–464 aa, EGFP-cyclinA1). For the pEGFP-C3-PDZ-cyclin A1 (EGFP-PDZ-cyclinA1) construct, cyclin A1 gene was inserted into the *Eco*RI-*Sal*I sites of pEGFP-C3-PDZ, which was cloned into pEGFP-C3 vector by the L-periaxin PDZ domain [18 to 102 amino acids (aa)]. The following primers were used: L-PRX-F: 5′-CTGAATTCAGCCCCAACGCGACACCTC-3′, L-PRX-R: 5′-GTGTCGACGACAGCCGCAGCCTG-3′; DPDZ-L-PRX (104 to 1398 aa)-F: 5′-GTGAATTCACCGGGGACCTGGCTCTG-3′, DPDZ-L-PRX (104 to 1398 aa)-R: 5′-GTGTCGACGACAGCCGCAGCCTG-3′; PDZ (18 to 102 aa)-F: 5′-GTGGATCCTTGGTGGAAATTATCGTG-3′, PDZ (18 to 102 aa)-R: 5′-CGGAATTCCACAGTGCGCTTCAGGC-3′; cyclin A1 (NM_001111045, 1 to 464 aa)-F: 5′-ACGAATTCATGGAGACCGGCTTTCCCGCA-3′, cyclin A1-R: 5′-CGGTCGACTTGTAGAAGAAGAACTGC-3′.

### Site-directed mutagenesis

The *Fast* Mutagenesis System (Transgene, Beijing, China) was used to create PDZ NES mutants. We generated each PCR products with the following conditions according to the manufacturer's instructions: 95°C for 5 min; 30 cycles of amplification (94°C for 1 min, 62°C for 30 s, and 72°C for 7 min); and 72°C for 10 min. The amplified products were digested using the restriction endonuclease *Dpn*I to eliminate the methylated and hemimethylated parental DNA template. The reaction mixture was transformed into *E.coli*XL-10 ultracompetent cells. Double-stranded plasmid DNA was purified from the transformants by using a plasmid mini kit (Omega, United States). Clones bearing each of the desired mutations were determined by DNA sequence analysis. The following primers were used for site-directed mutagenesis amplification:

L_83,85_/Q up: 5′-TTCAAGTACGAGGACGCACAACGCCAGCTGCAATGC-3′;

L_83,85_/Q dn: 5′-TGGCGTTGTGCGTCCTCGTACTTGAAGTTCTCGAA-3′.

### Cell culture and Transfection and Leptomycin B (LMB) treatment

Rat RSC96 Schwann cells (Type Culture Collection of the Chinese Academy of Sciences, Shanghai, China) were maintained in Dulbecco's modified Eagle's medium supplemented with 10% fetal bovine serum at 37°C in a humidified atmosphere containing 5% CO_2_. RSC96 cells were seeded on sterile glass cover slips in six-well trays and cultured overnight prior to transfections. The cells were transiently transfected with TurboFect transfection reagent (Thermo Scientific, United States) at 50% to 70% confluency with 4 μg of DNA and 6 μL of transfection reagents per well. After 24 h post-transfection, the cells were washed with phosphate-buffered saline (PBS) and processed for microscopic analysis. The cells were washed twice with PBS and incubated with LMB (Beyotime Institute of Biotechnology, China) at a final concentration of 37 nmol/L for the indicated period.

### Microscopic analysis

EGFP fusion proteins in the cells were visualized using Delta Vision (API, United States). The cells were washed twice with PBS and fixed with ice-cold methanol for 5 min at 4°C. Nuclear DNA was stained with 1 μg/ml DAPI (Solarbio, Beijing, China) for 15 min at room temperature. Subsequently, the cells were washed with PBS and mounted on microscope slides by using Vectashield reagent at 24 h after transfection.

### Cell harvesting and fractionation

Transfected with TurboFect transfection reagent (Thermo Scientific, United States) at 50% to 70% confluency and treated with or without LMB, the cells were harvested by centrifugation and washed with PBS. The cells were pelleted by centrifugation, resuspended in 350 μl buffer A (20 mM HEPES [pH 7.5], 10 mM KCl, 0.1 mM EDTA, 0.1 mM EGTA, 1 mM dithiothreitol, and protease and phosphatase inhibitors), and incubated on ice for 15 min. Cells were lysed by addition of 10% NP-40 to a final concentration of 1% and vortexed for 1 min, then centrifuged for 10 min at 14,000 rpm at 4°C. The supernatant cytosolic fraction was transferred to a new tube, and the nuclear pellet was washed with 400 μl buffer A and pelleted for 10 min at 14,000 rpm at 4°C. Nuclei were solubilized by addition of one pellet volume of NE buffer (20 mM Tris [pH 8.0], 420 mM NaCl, 1.5 mM MgCl_2_,0.2 mM EDTA, 25% glycerol, and protease and phosphatase inhibitors), and one-fourth pellet volume of 5 M NaCl.The soluble nuclei fraction solution was obtained by centrifugation for 10 min at 14,000 rpm at 4°C [17].

### Western blot

80 μg of above proteins were separated in a 10% SDS-PAGE gel and transferred onto a PVDF membrane (Millipore, United States). The membrane was rinsed in Tris-buffered saline (TBS) with 0.1% Tween-20 (TBST) and blocked with 5% fat free milk in TBST at room temperature for 1 h. The membrane was then incubated with anti-GFP (1∶1000 dilution;Transgene, Beijing, China), anti-β-actin, anti-Histone antibody (1∶1000 dilution; Sangon Biotech, China) followed by incubation with horseradish peroxidase-conjugated secondary antibodies (1∶1000 dilution; Transgene, Beijing, China). The immunoblots were detected by enhanced chemiluminescence reaction (Engreen Biosystem, China) and measured with densitometry.

### Sequence alignment

Multiple sequence alignment was performed in Clustal ×1.81 and adjusted to account for the conserved hydrophobic positions.

## Results

### Subcellular localization of the full-length L-periaxin and its deleted mutants

**Figure 1:**
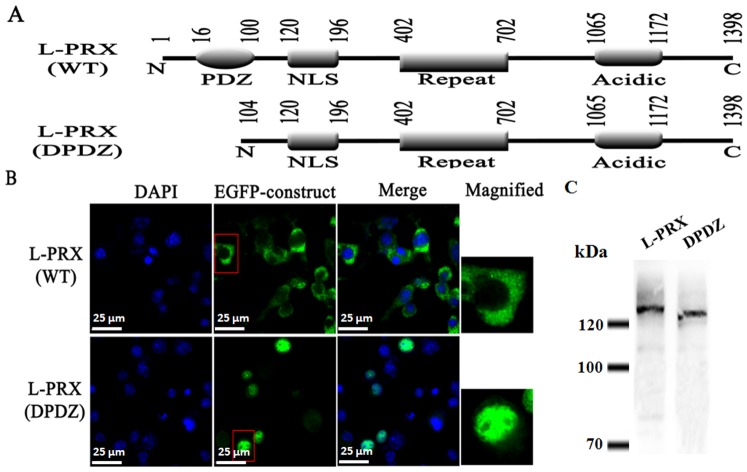
PDZ is necessary to accomplish an efficient nuclear export of L-PRX. (A) Structures of L-PRX and its PDZ-deleted mutants (DPDZ-L-PRX). Amino acid residues flanking each domain are numbered on top of the diagram. (B) Subcellular localization of EGFP-tagged L-PRX and its PDZ-deleted mutant (DPDZ-L-PRX). RSC96 cells were transfected with the plasmids encoding EGFP-tagged full-length L-PRX and its DPDZ-L-PRX. Scale bar = 25 μm. (C) GFP fusion constructs detected by Western blotting to ensure that the visualized proteins are not free GFP, or GFP fused to a truncated protein.

The expression plasmids encoding the full-length L-periaxin (L-PRX) and the mutant without a PDZ domain (DPDZ-L-PRX) were constructed (Fig. 1A). To ensure that the visualized proteins are not free GFP, or GFP fused to a truncated protein, Western blotting was performed to detect all the GFP fusion constructs (Figure 1C). All of the constructs were tagged at the N-terminus with an EGFP to detect expression products under a Delta Vision microscope. The RSC96 cells transfected with these plasmids were subjected to fluorescence analysis and revealed that L-PRX was predominantly localized in the cytoplasm (Fig. 1B). By contrast, DPDZ-L-PRX was expressed in RSC96 cells and mainly localized in the nucleus (Fig. 1B). The PDZ domain was probably involved in the cytoplasmic localization of L-periaxin because the mutant lacked a region (residues 18–102) containing a PDZ domain.

### Cytoplasmic localization of cyclin A1 fused with the PDZ domain of L-periaxin

**Figure 2:**
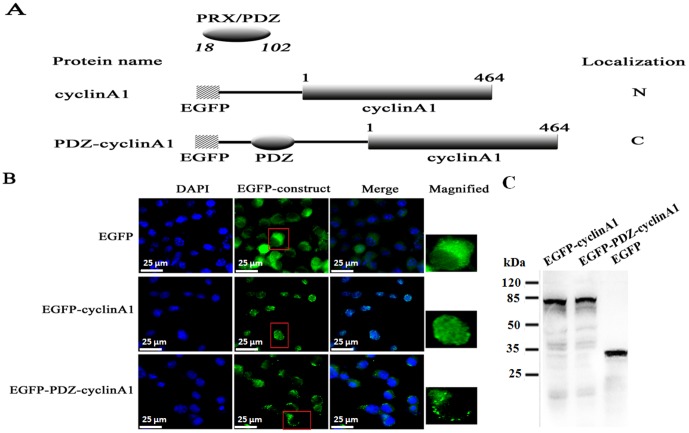
Cytoplasmic localization of a normal nuclear cyclin A1 by fusing with the PDZ domain of L-periaxin. (A) Schematic representation of cyclin A1 and its chimeric proteins fused with the PDZ domain of L-periaxin. N-terminal hatched boxes indicate EGFP-tagged peptides. The plain numbers on top of the boxes indicate the amino acid residue number of cyclin A1. Italic numbers correspond to the residues flanking the PDZ-domain of L-periaxin. The subcellular localization of each protein, when expressed in RSC96 cells, is indicated on the right. N, nucleus, C, cytoplasm. (B) Fluorescence analysis of RSC96 cells transfected with the plasmids encoding EGFP, EGFP-cyclin A1, and EGFP-PDZ-cyclin A1. Scale bar = 25 μm. (C) GFP fusion proteins are stable in the cell which is shown by Western blotting.

To determine whether or not the PDZ domain of L-periaxin can localize in the cytoplasm of a nuclear protein, we constructed plasmids encoding the following EGFP-tagged proteins: cyclin A1 and a cyclin A1 chimeric protein with the PDZ domain of L-periaxin (Fig. 2A). Western blotting results showed that EGFP- cyclin A1 and EGFP-PDZ-cyclin A1 fusion proteins were stable in the cell (Fig. 2C). Cyclin A1 is predominantly located in the nucleus throughout the cell cycle [18] and localized in the cytoplasm by fusing with a cytoplasmic localization signal of cyclin B1 [19]. Fluorescence was detected in the cells transfected with these plasmids, revealing that PDZ-cyclin A1 was localized in the cytoplasm, whereas the control cyclin A1 was localized in the nucleus (Fig. 2B). Thus, fluorescence analysis provided evidence that the PDZ domain of L-periaxin could induce the localization of the nuclear cyclin A1 in the cytoplasm.

### Nuclear export of L-periaxin is inhibited by LMB

**Figure 3:**
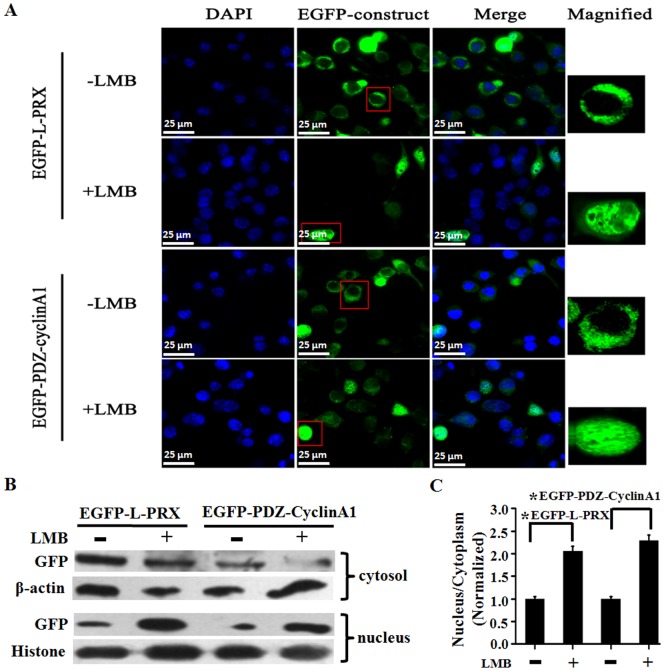
Nuclear export of L-periaxin is inhibited by LMB. (A) RSC96 cells were transfected with the plasmids encoding EGFP-tagged L-PRX and PDZ-cyclin A1. The cells in the second and fourth panels were treated with 37 nmol/L LMB for 1 h after 7 h post-transfection. EGFP-L-PRX and EGFP-PDZ-cyclin A1 localization was observed by Delta Vision. Scale bar = 25 μm. (B) The cytosol and nucleus were separated and determined the distribution of protein in the two fractions by Western blotting. β-actin and Histone served as an internal control for cytosol and nucleus, respectively. (C) Quantitative analysis of the distribution of EGFP fusion proteins in the two fractions by normalized to the internal control level. *P<0.05.

LMB is an antifungal compound that potently and specifically prevents NES-dependent nuclear protein export by inhibiting the interaction of NES with the export receptor chromosome region maintenance 1 (CRM1)/exportin [20]. Therefore, we used LMB in this study to determine whether or not the same mechanism mediates the release of L-periaxin from the nucleus. Fig. 3A shows that LMB blocked the export of L-periaxin and PDZ-cyclin A1 (second and fourth panels). The cytoplasm and nucleus were separated and determined the distribution of EGFP-L-periaxin and EGFP-PDZ-cyclin A1 in the two fractions by Western blotting. The results indicated that LMB treatment reduced the distribution of EGFP-L-periaxin and EGFP-PDZ-cyclin A1 in the cytoplasm (Fig.3B and 3C). Therefore, the nuclear export of L-periaxin occurs via a process sensitive to LMB that requires an NES sequence present in L-periaxin PDZ domain.

### Nuclear export of L-periaxin is mediated by a leucine-rich NES

To identify the sequence motifs that possibly mediate the binding of L-periaxin-PDZ domain to the export receptor CRM1, we applied a two-step approach: *in silico* prediction of candidate NES (cNES) and predicted cNES experiment.

**Figure 4:**
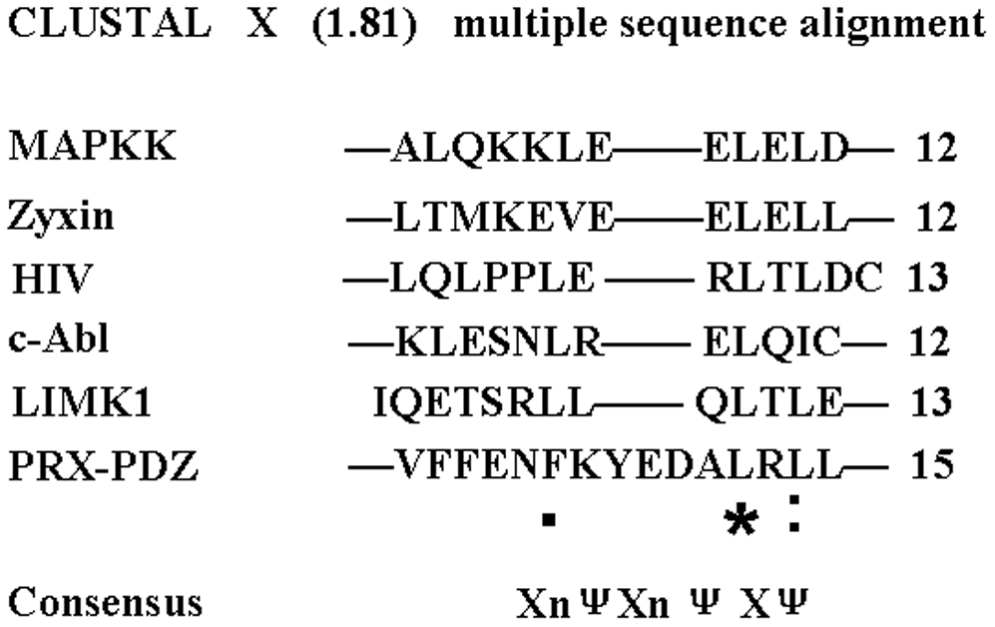
Alignment of potential NES sequences of L-periaxin with previously characterized leucine-rich NESs. NES sequences in mitogen-activated protein kinase kinase (MAPKK), zyxin, HIV.Rev, c-Abl, and LIM-kinase 1 are obtained from previous studies [22], [29], [30], [31], [32]. Residue numbers are indicated in parentheses. Consensus sequence is shown at the bottom. Ψ indicates hydrophobic residues, which include leucine, isoleucine, valine, phenylalanine, and methionine.

NES is characterized by a short peptide sequence enriched in regularly spaced hydrophobic residues defined as ΨX_2–3_ΨX_2–3_ΨXΨ, where Ψ is leucine, isoleucine, valine, phenylalanine, or methionine and X is any amino acid [21]. The primary amino acid sequence of the L-periaxin PDZ domain was analyzed using a web-based NES prediction program: NetNES [21]. An NES was identified in the amino acid sequence of L-periaxin PDZ domain between residues 73 and 86 by using the NetNES program. Known “leucine-rich” NESs (mitogen-activated protein kinase kinase (MAPKK), zyxin, HIV.Rev, c-Abl, LIM-kinase 1) were compared with the sequences found in L-periaxin PDZ domain. The results indicated the presence of leucine-rich stretches with the consensus X_n_ΨX_n_ΨXΨ (where Ψ corresponds to hydrophobic residues and X corresponds to any amino acid), which is similar to the sequences that function as NES in numerous proteins [21]. In this regard, the amino acid sequence (VFFENFKYEDALRLL) between residues 73 and 86 in the L-periaxin PDZ domain is significantly similar to the NES sequences reported for MAPKK, zyxin, HIV.Rev, c-Abl, and LIM-kinase 1, sharing underlined hydrophobic (leucine) properties (Fig. 4).

**Figure 5:**
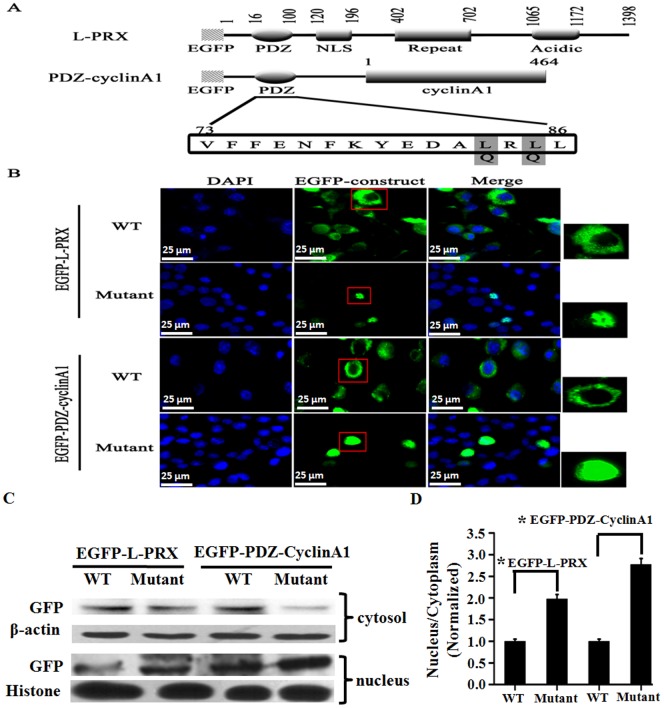
Nuclear accumulation of L-periaxin by mutating Leu_83_ and Leu_85_ to Gln. (A) Structures of L-PRX, PDZ-cyclin A1, and their mutants. Gray boxes indicate the amino acid residues replaced with Gln in the PDZ domain. (B) RSC96 cells were transfected with the plasmids encoding EGFP-tagged L-PRX, PDZ-cyclin A1, and their mutants. Scale bar = 25 μm. (C) The cytoplasm and nucleus were separated and determined the distribution of protein in the two fractions by Western blotting. β-actin and Histone served as an internal control for cytosol and nucleus, respectively. (D) Quantitative analysis of the distribution of EGFP fusion proteins in the two fractions by normalized to the internal control level. *P<0.05.

To determine whether or not the NES in the same PDZ domain contributes to the cytoplasmic localization of L-periaxin, we transfected the mutants of full-length L-PRX and PDZ-cyclin A1, namely, L-periaxin (L_83,85_/Q) and PDZ-cyclin A1 (L_83,85_/Q), respectively [in which two hydrophobic residues (Leu-83 and Leu-85) in NES were replaced with glutamine to destroy the nuclear-export activity of NES], to RSC96 cells. We then examined the localization of these mutants in the transfected cells. In contrast to the wild-type L-periaxin and PDZ-cyclin A1, L-periaxin (L_83,85_/Q) and PDZ-cyclin A1 (L_83,85_/Q) were localized mainly in the nucleus (Fig.5B), and the distribution of EGFP-L-periaxin and EGFP-PDZ-cyclin A1 in the cytoplasm were reduced in L-periaxin (L83,85/Q) and PDZ-cyclin A1 (L83,85/Q) groups (Fig.5C and 5D). These results indicated that NES was required for the cytoplasmic localization of L-periaxin. NES also exports the full-length L-PRX from the nucleus (Fig. 5).

## Discussion

In this study, we demostrated that PDZ domain of L-periaxin might play an important role in its translocation from the nucleus to cytoplasm. L-periaxin is targeted to the nucleus of embryonic Schwann cells and redistributed to plasma membrane processes of the myelinating Schwann cell, where it is believed to functioning in signaling complex. There are at least two possible mechanisms to localize the protein in the cytoplasm, a cytoplasmic retention mechanism by which the protein is tethered to particular cytoplasmic anchor proteins, and a nuclear export mechanism by which the protein is forcibly excluded from the nucleus to the cytoplasm [22]. We speculated whether L-periaxin should aslo possesses nuclear export signal (NES) that could allow it to shuttle from the nucleus to cytoplasm. Some PDZ domain proteins, such as Zo-1, Zo-2, MAGI-1c, and LIM-kinase 1, can be redistributed between the cell cortex and the nucleus. This event possibly demonstrates their function to transmit regulatory signals between the cell surface and the nucleus [22], [23], [24]. For example,tight junction protein ZO-2 contains four putative NES, in which two are located at the second PDZ domain (NES-0 and NES-1) and the other two are found at the GK region (NES-2 and NES-3) [25]. LIM-kinase 1 contains two functional leucine-rich NESs in its PDZ domain [26]. Although the reason for such redistribution is unknown, this finding is possibly considered as a response to the movement of these scaffolding proteins into and out of the nucleus.

The present study showed that L-periaxin contained an NES similar to previously characterized leucine-rich NESs in the PDZ domain [25], [26]. The nuclear export activity of L-periaxin can be inhibited by malfunctional NES mutation or by LMB treatment that can induce changes in the localization of L-periaxin from the cytoplasm to the nucleus. This finding indicated that the predominantly cytoplasmic localization of L-periaxin, under normal conditions, is crucially supported by this NES.

Although it is not yet clear what the interaction net of L-periaxin might be, L-periaxin is included in the list of proteins redistributed in the nucleus as well as in cortical signaling and adhesion complexes. Based on its localization to the Cajal bands of Schwann cells and the dymyelinating neuropathy phenotype associated with the gene knockout mouse model,periaxin has been confirmed to play an essential role in stabilization of the Schwann cell-axon unit [27]. We suppose that nuclear targeting of L-periaxin in embryonic Schwann cells may play any role in nulear or sequester L-periaxin from inappropriate interactions in the cytoplasm untill the correct ligand becomes available at the cell cortex of the maturing myelin forming Schwann cell. Once ligand, such as dystrophin-related protein 2 (DRP2) can be available, L-periaxin will be interact with DRP2 to provide a link between the extra celluar matrix and Schwann cell by CRM1-dependent nuclear export [9]. Perixain is the only protein in the database that shares a significant degree of homology with the repeated domain of AHNAK [28]. Like AHNAK,L-periaixn might be transmitting signals from the cell periphery to the nucleus or even physically transferring proteins to the required locales within the cells. The stimulus or signal transduction that influences the nucleocytoplasmic distribution of L-periaxin should be a further research.
